# Fasting cycles potentiate the efficacy of gemcitabine treatment in *in vitro* and *in vivo* pancreatic cancer models

**DOI:** 10.18632/oncotarget.4186

**Published:** 2015-05-19

**Authors:** Martina D'Aronzo, Manlio Vinciguerra, Tommaso Mazza, Concetta Panebianco, Chiara Saracino, Stephen P. Pereira, Paolo Graziano, Valerio Pazienza

**Affiliations:** ^1^ Gastroenterology Unit, I.R.C.C.S. “Casa Sollievo della Sofferenza” Hospital San Giovanni Rotondo (FG), Italy; ^2^ Institute for Liver and Digestive Health, Division of Medicine, University College London (UCL), London, United Kingdom; ^3^ School of Science and Technology, Nottingham Trent University, Nottingham, United Kingdom; ^4^ Bioinformatics Unit I.R.C.C.S. “Casa Sollievo della Sofferenza”, Istituto Mendel, Italy; ^5^ Pathology Unit I.R.C.C.S. “Casa Sollievo della Sofferenza” Hospital San Giovanni Rotondo (FG), Italy

**Keywords:** pancreatic cancer, gemcitabine, hENT1

## Abstract

**Background/aims:**

Pancreatic cancer (PC) is ranked as the fourth leading cause of cancer-related deaths worldwide. Despite recent advances in treatment options, a modest impact on the outcome of the disease is observed so far. Short-term fasting cycles have been shown to potentiate the efficacy of chemotherapy against glioma. The aim of this study was to assess the effect of fasting cycles on the efficacy of gemcitabine, a standard treatment for PC patients, *in vitro* and in an *in vivo* pancreatic cancer mouse xenograft model.

**Materials and Methods:**

BxPC-3, MiaPaca-2 and Panc-1 cells were cultured in standard and fasting mimicking culturing condition to evaluate the effects of gemcitabine. Pancreatic cancer xenograft mice were subjected to 24h starvation prior to gemcitabine injection to assess the tumor volume and weight as compared to mice fed *ad libitum*.

**Results:**

Fasted pancreatic cancer cells showed increased levels of equilibrative nucleoside transporter (hENT1), the transporter of gemcitabine across the cell membrane, and decreased ribonucleotide reductase M1 (RRM1) levels as compared to those cultured in standard medium. Gemcitabine was more effective in inducing cell death on fasted cells as compared to controls. Consistently, xenograft pancreatic cancer mice subjected to fasting cycles prior to gemcitabine injection displayed a decrease of more than 40% in tumor growth.

**Conclusion:**

Fasting cycles enhance gemcitabine effect *in vitro* and in the *in vivo* PC xenograft mouse model. These results suggest that restrictive dietary interventions could enhance the efficacy of existing cancer treatments in pancreatic cancer patients.

## INTRODUCTION

Pancreatic cancer (PC) is ranked as the fourth leading cause of cancer related deaths worldwide [1]. Due to the absence of early symptoms, and to extreme aggressiveness and chemotherapy resistance of the tumor, PC is often diagnosed at an advanced stage of disease rendering current treatment options ineffective. Up to 80-90% of PC patients are not eligible for resection at presentation and the available therapeutic strategies based on conventional chemotherapy are still largely unsatisfactory considering that less than 5% will survive up to 5 years [2, 3]. Efforts are needed to find effective treatment or novel therapeutic approaches to overcome the resistance of PC to conventional anticancer therapies. A standard therapy for treatment of patients with PC with either curative or palliative intent is gemcitabine [4, 5]. The latter is a nucleoside analogue (similar to cytosine) with tumor growth arrest properties due to the two fluorines on the carbon 2′, instead of the hydrogen atoms, which render instability in the DNA chain during the replication process. Gemcitabine is taken up within pancreatic cancer cells primarily by human equilibrative nucleoside transporter 1 (hENT1) [6]. After being phosphorylated by DCK (deoxycytidine kinase) to its active form, it finally exerts its anti-tumor growth properties. Several studies analyzed the expression of hENT1 as it was expected to be predictive for clinical outcomes in pancreatic cancer patients treated with gemcitabine [7, 8]. Another target of gemcitabine is the human ribonucleotide reductase (RRM1), a key enzyme involved in the homeostasis of nucleotide pools affecting cell proliferation, migration and metastasis [9], which was found to improve survival in gemcitabine-treated patients displaying lower levels of RRM1, whereas higher levels did not [10, 11]. Recently, major health benefits associated with dietary restriction have been demonstrated, such as amelioration of cardiovascular diseases, diabetes, insulin resistance, immune disorders, slowing of the aging process and reduced risks of cancer [12]. Recent studies in rodent and *in vitro* models uncovered a potential link between short term starvation and improved efficacy of chemotherapy for some types of cancer [13, 14]. At present no data are available on the effect of short term starvation on pancreatic cancer. Here we sought to investigate whether fasting is able to improve chemotherapeutic efficacy in pancreatic cancer cells and in a PC xenograft mouse model.

## RESULTS

### Cell viability assay in fasted and non-fasted pancreatic cancer cells

**Figure 1:**
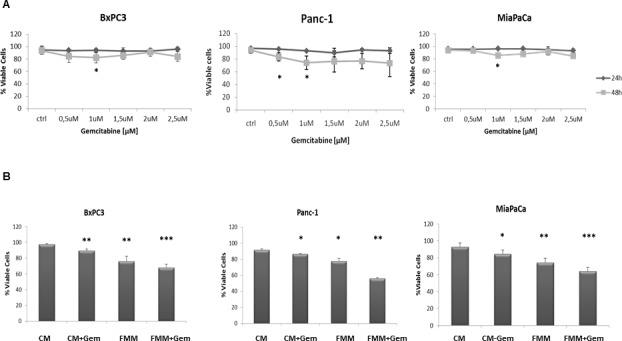
Cell viability assay BxPC-3, PANC-1 and MiaPaca-2 cells were treated for 24h and 48h with gemcitabine at a concentration range between 0.5 μM and 2.5 μM **A**. Cell viability assay was performed on cells growing on control (CM) or fasting mimicking medium (FMM) after 48h of gemcitabine treatment at a concentration of 1 μM, including untreated cells used as control samples **B**. Results are expressed as means ± SE. Differences were considered as significant when *P* < 0.05 (*) or *P* < 0.01 (**) or *P* <0.001 (***).

As a first step we performed a time and dose response curve in order to establish the effect of gemcitabine on the viability of three PC cell lines, BxPC3, Panc-1 and MiaPaCa-2. As shown in Figure 1A, 1μM of gemcitabine slightly reduced cell viability in all the cell lines and this concentration was used in all subsequent *in vitro* experiments. Of note, higher concentrations of gemcitabine did not affect cell mortality rate, most likely because higher-dose of gemcitabine treatment enriches chemotherapy resistant cells as already demonstrated [15]. When gemcitabine and fasting mimicking medium (FMM, 0.5g/L glucose and 1% FBS) treatments were combined, pancreatic cancer cells displayed the highest death rate compared to FMM or gemcitabine added to a control standard medium alone (CM, 2g/L glucose and 10% FBS) (Figure 1B).

### Fasting inhibits cell migration

**Figure 2:**
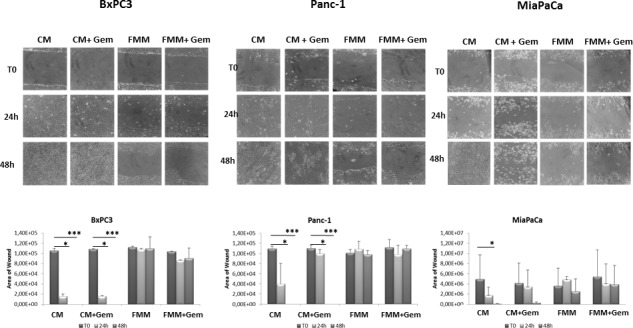
Wound healing assay Cell migration of BxPC3, PANC-1 and MIAPaCa-2 upon treatment with gemcitabine alone (1μM) or in combination with fasting mimicking medium **A**. The area of wound was measured for all the fields of each well using Image J **B**.

To investigate the effect of fasting on pancreatic cancer cell migration, a key event in carcinogenesis, we performed an *in vitro* wound-healing assay. Gemcitabine in combination with FMM significantly reduced cell migration of BxPC3, PANC-1 and MIAPaCa-2 while gemcitabine treatment alone failed to do so (Figure 2A-2B). Remarkably, FMM alone was as effective as combined treatment in inhibiting cell migration (Figure 2A-2B).

### Effect of fasting on cell cycle

**Figure 3:**
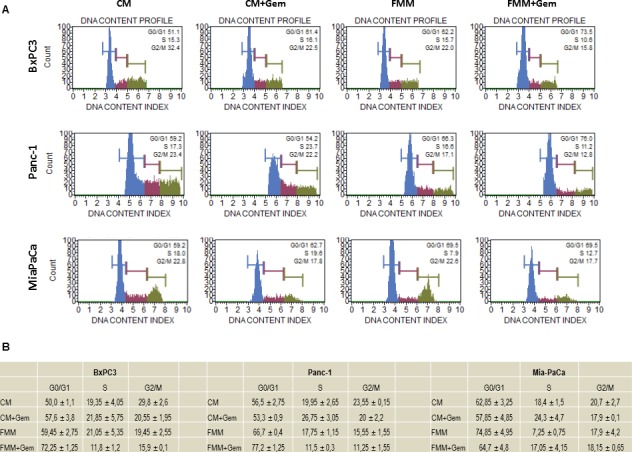
Cell cycle analysis BxPC3, PANC-1 and MIAPaCa-2 upon treatment with gemcitabine alone (1μM) or in combination with fasting mimicking medium were subjected to cell cycle analysis using the Muse Cell Analyzer **A**. Table in panel **B** shows the quantitative measurements reported as means ± SE.

Cell cycle derangement is one of the main tumor arrest properties of gemcitabine [16]. We performed a cell cycle analysis to assess the effect of gemcitabine alone or in combination with FMM on PC cells as compared to PC cells cultured in CM. Figure 3 shows that PC cells treated with gemcitabine in CM condition displayed a slight but non-significant increase in G0/G1 phase while combined treatment (fasting plus gemcitabine) significantly increased the percentage of PC cells in G0/G1 phase, with decreased S phase (synthesis) and G2/M phase in BxPC3 and PANC-1 cells. Although the percentage of cells in G0/G1 phase was increased upon combined treatment in MIAPaCa-2 cells, the latter did not reach statistical significance.

### Fasting augments hENT1 and decreases RRM1 expression

**Figure 4:**
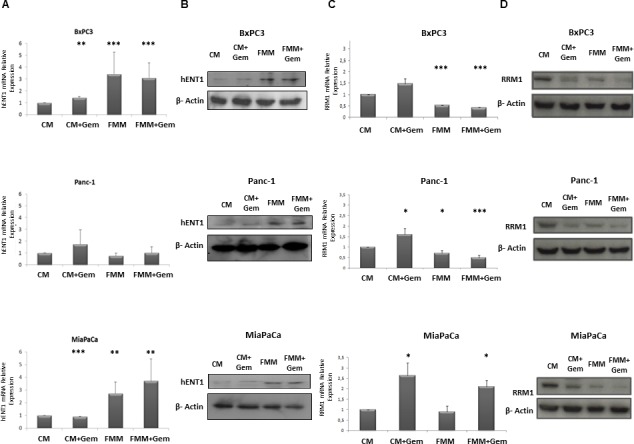
hENT1 mRNA and protein expression by qRT-PCR and immunoblot (column A and column B) in control PC cells and treated with fasting +/− gemcitabine. RRM1 mRNA and protein expression by qRT-PCR and immunoblot (column **C** and column **D**) in control PC cells and treated with fasting +/− gemcitabine.

To better understand the mechanism through which fasting was more effective than control media, we hypothesized that the low glucose level contained in the fasting medium could be responsible for the activation of the nucleoside transporter protein (hENT1) as reported in other studies [17-20], potentiating the gemcitabine effect in inhibiting RRM1 expression. As shown in Figure 4A, hENT1 mRNA expression increased upon exposure of BxPC-3 and MIAPaCa-2 cells to FMM and FMM plus gemcitabine while no significant changes were observed in PANC-1 cells. At the protein level, hENT1 increased in all cell lines when subjected to FMM (Figure 4B). Furthermore, FMM and FMM plus gemcitabine significantly reduced RRM1 mRNA levels in BxPC3 and Panc-1 cells, but not in MIAPaCa-2 (Figure 4C), whilst RRM1 protein expression was reduced in all cell lines as compared to controls (Figure 4D).

### Fasting increases gemcitabine uptake

**Figure 5:**
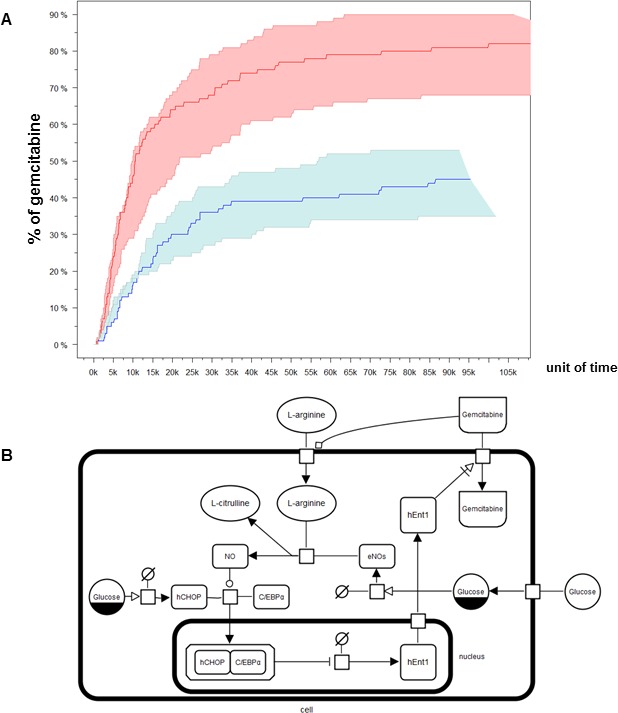
Modeling and simulation of Gemcitabine uptake Several SBGN glyphs represented distinct entities (nucleic acids, macromolecules and complexes) and processes (transport, modulation, stimulation, catalysis and complex formation) **B**. Plots of temporal concentration changes of Gemcitabine within the cell. Min/max/mean stochastic realizations are drawn for control diet (blue) and fasting (red) **A**.

In human endothelial cells high glucose leads to increased synthesis of nitric oxide (eNOS) and reduced uptake of adenosine-like molecules (such as gemcitabine) through a reduced expression and activity of human hENT1 [18] which is thought to be mediated by the transcription factor hCHOP–C/EBPα complex [18]. According to this model, glucose molecules interfere with hENT1 transcription through the enhancement of eNOS, resulting in hCHOP-C/EBPα transcription complex formation and shuttling to the nucleus (Figure 5B). To unravel the indirect relationship between fasting and gemcitabine uptake rates in PC cells we adopted stochastic modeling. Varying the concentration of glucose from 2g/L (CM regimen) to 0.5 g/L (FMM regimen) and considering a concentration of gemcitabine of 1μM, we drew stochastically the quantitative evolution of gemcitabine uptake for a maximum of 115 thousands of simulated units of time. To guarantee solid confidence intervals, we simulated the modeled system under both diet regimes a thousand times and monitored the temporal concentration changes of gemcitabine within the cell. We then verified that the CM medium contributed to a mean gemcitabine uptake of 40%, while FMM medium more than doubled (82.3%) its mean transport rate (Figure 5A).

### Fasting potentiates gemcitabine effect in a PC xenograft mouse model

**Figure 6:**
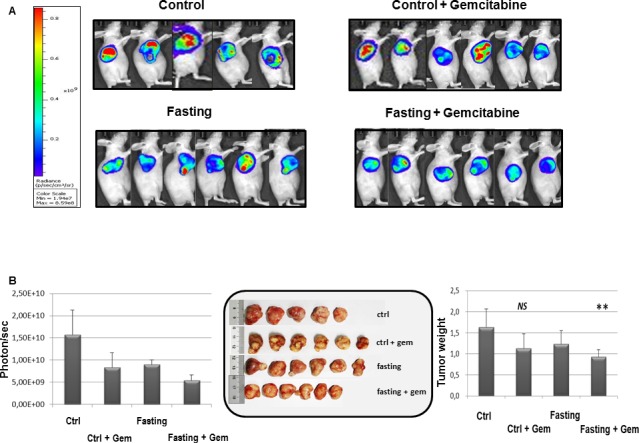
Effect of fasting on PC tumor When tumor size reached an average volume of 100 mm^3^, BxPC-3-luc tumor-bearing nude mice were randomly assigned into 4 groups and started dosing immediately. Group 1 (given normal saline, i.p, qw), group 2 (gemcitabine, 100 mg/kg, i.p, qw), group 3 (the mice in this group were fasted 24h before given normal saline, i.p, qw), group 4 (the mice in this group were fasted 24h before given gemcitabine, 100mg/kg, i.p, qw). Bioluminescence signaling measured as photons/sec **A**. The tumor masses were harvested, photographed and weighed **B**.

We then evaluated the effects of combined fasting and gemcitabine treatment in a xenograft pancreatic cancer mouse model. As shown in Figure 6 mice subjected to 24h of complete fasting before gemcitabine injection displayed a significant retarded progression of pancreatic cancer tumor (*p =* 0.04). Notably, fasting in the absence of chemotherapy was as effective as gemcitabine alone, although this was just below the statistical significance.

### hENT1, RRM1, Ki67 and BCL-2 expression in pancreatic cancer biopsies of mice under fasting condition

**Figure 7:**
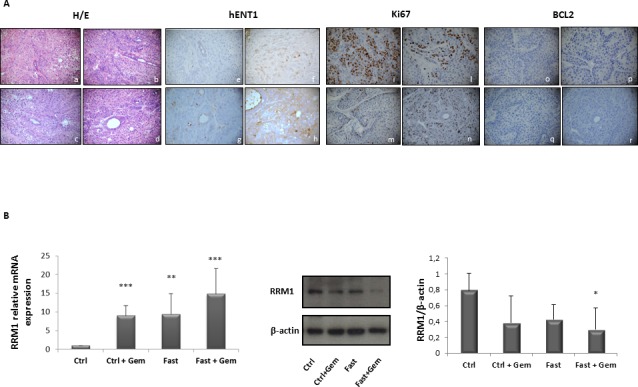
Immunoistochemical evalutation of hENT1, Ki67 and BCL-2 expression in PC biopsies of mice allocated in to the 4 different groups Representative H&E pictures, hENT1, Ki67 and BCL-2 immunohistochemical expression of pancreatic sections from control (a-e-i-o) gemcitabine treated (b-f-l-p), fasted (c-g-m-q) and fasted plus gemcitabine (d-h-n-r) treated mice (40X magnification) **A**. hENT1, Ki67 and BCL-2 immunoreactivity was evaluated in blind using a semiquantitative scoring system in ten high power fields (10HPF, X 400) according to a semiquantitative scale (−: 0%; +: 1-33%; ++: 34-66%; +++: 67-100%). RRM1 mRNA and protein expression levels measured by qRT-PCR and by immunoblot respectively in control, gemcitabine treated, fasted and fasted plus gemcitabine treated mice **B**.

Since a potential prognostic role for hENT1 and for RRM1 has been postulated [9, 21], and increased hENT1 levels enhance the response to gemcitabine in human pancreatic cancer [19] and are associated with a longer survival [6, 22-24], we then assessed hENT1 protein expression in pancreatic cancer biopsies of the nude mice allocated in the four treatment groups. In Figure 7A it is shown that hENT1 expression was more prominent in PC mice subjected to combined fasting and gemcitabine treatment as compared to control mice. 5 out of 6 (83%) mice subjected to 24h of complete fasting prior to gemcitabine injection (Figure 7A panel h) displayed positive levels of hENT1 as compared to mice allocated in gemcitabine (f) and fasting alone (g) groups (50%). 60% of control mice (panel e) showed negative hENT1 expression whilst the remaining 40% showed a weak signal. As for RRM1, an inverse correlation between RRM1 mRNA and protein levels was found: tissue samples from pancreatic cancer biopsies of the nude mice with higher levels of RRM1 mRNA (mice treated with gemcitabine, fasting or fasting plus gemcitabine treatment) displayed lower levels of the protein (Figure 7B), suggesting the existence of a post-transcriptional feedback mechanism within xenograft tumor between mRNA and protein levels of RRM1. Additionally we investigated the expression of markers of proliferation and cell death/apoptosis in pancreatic cancer biopsies from mice. As reported in Figure 7A, Ki67 positivity was higher in the control group (panel i) with 60% of mice displaying the highest positive level for Ki67 while 40% of mice in the gemcitabine group (panel l) and only 16% of mice in the fasting group (panel m) were positive for Ki67 staining. In the fasting plus gemcitabine group, all mice displayed intermediate levels of Ki67 (panel n). On the other hand BCL-2 expression was undetectable in all pancreatic cancer biopsies (panel o, p, q, r).

### Effects of fasting on the mTOR pathway in tumor samples

**Figure 8:**
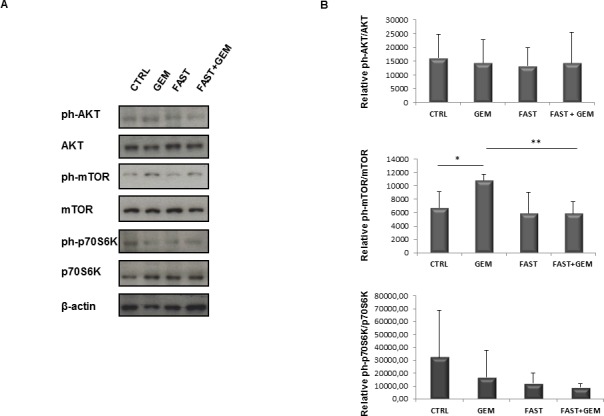
Immunoblot detection of AKT, ph-AKT(Ser473), mTOR, ph-mTOR(Ser2481), p70S6K, ph-p70S6K(Thr389) in tumor samples **A**. of control, gemcitabine treated, fasted and fasted plus gemcitabine treated mice. Quantitative measurement of proteins associated signal by densitometry **B**.

The protective effect of fasting may in part be due to the inhibition of the nutrient-sensing mTOR pathway in normal cells and also *in vitro* “fasting” and rapamycin protect normal cells and increase cytotoxicity in cancer cells [25, 26]. For this reason we investigated Akt and mTOR activity in pancreatic cancer mice' biopsies. As shown in Figure 8, no changes were observed in the activity of Akt, whereas significant changes were found in mTOR activity. In detail, gemcitabine treatment alone caused a significant increased phosphorylation levels of mTOR, which was abolished when combined with fasting. As concerns the downstream effector of mTOR, namely p70S6K, a trend towards a decrease in activity, without reaching statistical significance, was observed in all three groups gemcitabine, fasting, and fasting plus gemcitabine.

## DISCUSSION

Over the last four decades only small improvements in survival have been achieved for patients with pancreatic cancer, which represents one of the most aggressive cancers due to its therapeutic resistance [27]. This can be partly attributed to the ineffectiveness of chemotherapeutic compounds reaching the cancer cells, as suggested by previous preclinical and clinical work [28-30]. Dense stroma [31, 32] and deregulated cellular transport proteins [7] are considered pathological features constituting physical barriers to effective drug delivery. In fact, Koay et al, demonstrated that tumors displaying higher stromal scores had lower gemcitabine DNA incorporation [28]. Other studies showed that the gemcitabine transporter protein hENT1 is associated with the outcome of the disease [32].

Fasting has been practiced for millennia, but, studies have only recently shed light on its role in adaptive cellular responses that reduce oxidative damage and inflammation, achieving cellular protection [33]. Fasting, short-term calorie or protein-restricted diets have been reported to have beneficial effects in mice models of certain types of cancer [34, 35] accompanied by a decrease of side effects of chemotherapy in patients [36, 37]. Given that high glucose levels reduce hENT1 expression and increase pancreatic cancer cell proliferation [17-20], we investigated the effect of fasting on gemcitabine efficacy *in vitro* and in an *in vivo* pancreatic cancer mouse xenograft model.

Our data show that fasting not only increases hENT1 expression but is also able to increase gemcitabine uptake, as demonstrated by computational modeling and stochastic simulation, suggesting that fasting reduces tumor mass increasing gemcitabine delivery through hENT1 expression and repressing RRM1 protein. Moreover, we found that fasting affects mTOR activity, which plays a major role in maintaining the malignancy properties of pancreatic cancer stem cells [38]. In tumour biopsies, mTOR activation increased upon treatment with gemcitabine and returned to control levels upon fasting. The increase in mTOR phosphorylation upon gemcitabine treatment fits with previous finding of mTOR activation in gemcitabine-resistant pancreatic cells [39], and the reversal of this effect in fasting plus gemcitabine combined treatment may reflect the role of fasting in overcoming resistance and enhancing gemcitabine efficacy.

Our *in vitro* results show that FMM inhibits cell migration and shifts cell cycle into the G0/G1 phase while fasting cycles inhibit Ki67 *in vivo*. Our results are consistent with a recent study showing that calorie restriction decreases murine and human pancreatic tumor cell growth [40]. Besides gemcitabine, used alone or in combination, conventional drugs currently adopted to treat advanced PC and/or after surgical treatment include fluorouracil, irinotecan, cisplatin, and oxaliplatin. Notably, fasting alone tended to reduce tumor mass, which may represent an alternative for patients who are unable to undergo these conventional treatments. As for cancer prevention, no human data are available on the effect of fasting; however, its effect on IGF-1, insulin, glucose and ketone body levels could generate a protective environment that reduces DNA damage and carcinogenesis while at the same time creating hostile conditions for tumor and precancerous cells [33]. A strong mental discipline is needed by the patient to adhere to the fasting regimen, as underlined by Mathews and Liebenberg who propose as a potential solution the use of nutrient-haemodialysis to achieve fasting “mechanically” through dialysis [41]. Based on our *in vitro* results, in the frame context of pancreatic cancer, prolonged fasting (more than 24 hours) would be needed in order to achieve an increase in hENT1 expression to finalize the gemcitabine effect.

## MATERIALS AND METHODS

### Cell culture and fasting mimicking condition

BxPC-3, and PANC-1 cells were cultured either in control DMEM medium (CM) 2g/L glucose supplemented with 10% fetal bovine serum (FBS), 100 U/ml penicillin and 100 μg/ml streptomycin (Invitrogen Life Technologies, Milan, Italy) in 5% CO2 atmosphere at 37°C or in fasting mimicking medium (FMM) DMEM (0.5g/L glucose and 1%FBS). MIAPaCa-2 were maintained in control RPMI medium (Invitrogen Life Technologies, Milan, Italy) or in fasting mimicking condition RPMI medium as described elsewhere [14].

### Cell viability assay

The viability of cells was carried out performing a Trypan Blue Viability test at 24h and 48h upon gemcitabine treatment at a concentration range between 0.5 μM and 2.5 μM. The cells were trypsinized and resuspended in complete medium. Cell suspension was diluted 1:1 using a 0.4% Trypan Blue solution purchased by Sigma Aldrich. After one minute of incubation at room temperature, live and dead cells were counted using an hemocytometer (Biosigma) and a light microscope (Nikon Eclipse TS100), than the percentages of viable cell was calculated as (n° of cell alive/n° of total cells)x100.

### Wound healing assay

Cells were seeded into 6-well plate at a density of approximately 3×10^5^ and were cultured to confluence in control media for 48h and fasting media for further 24h. After that two perpendicular scratches were performed using 10μL filter tips, the media were replaced with control or Fasting media and the cell were treated with 1μM Gemcitabine. For each well three fields were chosen by marking with parallel lines on the external side of the plate. Images were taken for each field at 0h, 24h and 48h using a Canon Eos 40D. The area of wound was measured for all the fields of each well using Image J.

### Cell cycle analysis

Cells were harvested at least 3 hours before the experiment as already described [42]. After fixation with 1ml of 70% cold ethanol at −20°C, as indicated by the Muse Cell Cycle Kit User's Guide 200 μl of ethanol-fixed cells were incubated with propidium iodide and RNAse A for 30 minutes at room temperature, before loading on Muse Cell Analyzer (Millipore, Italy) according to the supplied staining protocol.

### Quantitative real-time polymerase chain reaction

Total RNA was extracted from plated cells using RNeasy Mini Kit (Qiagen, Milan, Italy) and subsequently treated with deoxyribonuclease I, according to the manufacturer's instructions. RNA concentration was assessed using Nanodrop spectrophotometer. Quantitative real time PCR for determining the expression levels of hENT1 was performed on 50 ng of purified RNA using the one step Quantifast SYBR Green RT PCR KIT (Qiagen) and the Human SYBR Green QuantiTect Primer Assay for SLC29A1 (QT000083) purchased from Qiagen. Reactions were set up in 96-well plates using a 7700HT Real-Time PCR System (Applied Biosystems, Foster City, CA), and all samples were assayed in triplicate. Optical data obtained were analyzed using the default and variable parameters available in the SDS software package (version 1.9.1; Applied Biosystems, Foster City, CA). Expression levels of target gene were normalized using the housekeeping control gene: TATA binding protein (TBP, QT00000721). mRNA amount of each target gene relative to TBP was calculated through the comparative Ct method, also called the 2(−ΔΔ^Ct^) method. Data are presented as the mean ± SE of at least three independent experiments.

### Immunoblotting

Total protein extraction from adherent cells and from snap frozen pancreatic cancer xenograft specimens was obtained using homemade Sample Buffer Leammli 2x (50 mM Tris–HCl, pH 6.8, 100 mM dithiothreitol, 2% sodium dodecyl sulfate, 0.1% bromophenol blue, 10% glycerol) supplemented with 2x protease inhibitor cocktail (COMPLETE; Roche Diagnostics, Mannheim, Germany), 1 mM phenylmethylsulphonyl fluoride and 1 mM sodium orthovanadate as already described [43] and through mechanical and detergent based lysis, Ripa buffer (150 mM NaCl, 50 mM tris HCl pH 7.4, sodium dodecyl sulfate (SDS) 0,1%, triton 1%, ethylenediaminetetraacetic acid (EDTA) 5 mM and 1% cholic acid sodium salt), supplemented with protease inhibitor cocktail (COMPLETE; Roche Diagnostics, Mannheim, Germany), 1 mM phenylmethanesulphonylfluoride and 1 mM sodium orthovanadate, respectively. The same amount of protein extract for each sample was loaded to 9% SDS-polyacrilammide gel and electroblotted on PVDF membrane (Whatman, Dassel, Germany) for 60 min at 60V. Membranes were incubated overnight at 4°C with primary antibody diluted 1:1000 into Blocking Buffer (1.25% Blotting Grade Biorad, 5% Sodium Azide in washing buffer) as previously reported [43]. Primary antibodies used were: rabbit polyclonal antibody hENT1 (H-115) (sc-134501), phospho AKT (sc-33437), mouse monoclonal antibody β-Actin (C4) (sc-47778) from Santa Cruz Biotechnology; antibodies against AKT(#9272), mTOR (#2972), phospho-mTOR (#2974), p70S6K (#9202), phospho-p70S6K (#9205) and RRM1 (#8637) were purchased from Cell Signaling. The membranes were washed three times with washing solution (1x Tris-Buffered Saline, 0.1% Tween 20 Sigma) and incubated for one hour at room temperature with appropriate secondary antibodies (BioRad, Hercules, CA goat anti-mouse and goat-antirabbit) diluted 1:3000. Membranes were washed several times with washing solution prior to detect the antigen-antibody complexes by enhanced chemiluminescence (ECL; Amersham Biosciences) with the signal detected on X-ray film (Amersham Biosciences) according to the manufacturer's instructions.

### Immunofluorescence

Cells were grown on coverslips and fixed by incubating for 10 minutes at room temperature with 4% paraformaldehyde. Subsequently cells were incubated for 2 minutes with 0.3% Triton X 100 to permeabilize cells. The coverslips were washed three times with Phosphate-Buffered-Saline solution (PBS) and incubated overnight at 4°C with the primary antibody rabbit polyclonal hENT1 (H-115) (sc-134501) diluted in PBS at ratio of 1:50. After three washes with PBS, secondary antibody incubation was carried out for 1h at room temperature using rhodamine labeled anti-rabbit antibodies (Jackson Lab) diluted at 1:100. Coverslips were washed again with PBS three times prior to be mounted on slides using Vectashield H1-200 (DBA Milan, Italy). A Nikon Eclipse E600 microscope was used for immunofluorescence analysis.

### Computational modeling and stochastic simulation

The hENT1 dynamics, as described in references [17, 18], was modeled in Systems Biology Graphical Notation (SBGN). The model focused on the transport of Gemcitabine within the cells. The key features of the model were the ability to represent both events like chemicals transport and reaction modulation, and species localization and compartmentalization. The SBGN model was translated into Systems Biology Markup Language (SBML), a simple and well known XML-based language, which adds components that reflect the natural conceptual constructs used by Systems Biology modelers [44]. Two semi quantitative models were obtained by adding information about the initial concentrations of the molecules constituting the two different media of the cells. These were then temporally simulated by Cyto-Sim: a formal language model and stochastic simulator of membrane-enclosed biochemical processes [45], in a computational parallel [46] environment yielding a thousand trajectories mimicking the Gemcitabine transport within the cell.

### Animal studies

We conducted our mouse work in an AAALAC (Association for Assessment and Accreditation of Laboratory Animal Care International) accredited experimental facility. Animal protocols were approved by the Institutional Animal Care and Use Committee (IACUC approval number is ANM13-001). 5-6 weeks old female Nu/Nu mice were maintained in a specific pathogen-free (SPF) environment throughout the experiments. BxPC-3-luc cancer cells were cultured and s.c. injected into Nu/Nu nude mice (right flank). A total number of 5×10^6^ tumor cells per mouse was suspended in 0.1 mL of PBS/matrigel mixture (1:1) and then injected. When tumor size reached an average volume of 100 mm^3^, BxPC-3-luc tumor-bearing nude mice were randomly assigned into 4 groups (6 mice/group) and started dosing immediately. Group 1 (Normal saline, i.p, qw), group 2 (Gemcitabine, 100 mg/kg, i.p, qw), group 3 (the mice in this group were fasted 24h before by giving normal saline, i.p, qw), group 4 (the mice in this group were fasted 24h before by giving Gemcitabine, 100mg/kg, i.p, qw). For fasting, mice were single caged and maintained in standard cages without access to food for 24 hours. Cages were changed immediately before the initiation of fasting cycle in order to avoid coprophagy or feeding on residual chow. Animals had free access to water. Gemcitabine was dissolved in saline (0.9% NaCl w/v in water) to generate a final concentration of 10 mg/mL. The drug was freshly dissolved before use, and the solution was homogeneous before injections. The i.p. injection volume was 100ul/10g mouse weight.

### Statistical analysis

Results are expressed as mean ± SE. Comparisons were made using Student's *t*-test. Differences were considered as significant when *P* < 0.05 (*) or *P* < 0.01 (**) or *P* < 0.001 (***).

### Immunohistochemistry

Formalin-fixed, paraffin-embedded pancreatic mice cancer sections allocated in the four different groups were immunostained by using commercially available detection kit (EnVision™ FLEX+, Dako, Glostrup, Denmark) following the manufacturer's protocol as previously described [47]. Primary antibody for hENT1 was purchased from Santacruz (cat. no. sc-134501) and diluted 1:75 while Ki67 (cat. no. M7240) and BCL2 (cat. no. M0887) were from Dako. The specificity of all reactions was checked replacing the primary antibody with normal serum alone. Positive and negative controls were used as appropriate and were run concurrently. hENT1 immunoreactivity was evaluated blindly by an expert pathologist (PG) assessing a semiquantitative scoring system in ten high power fields (10HPF, X 400) according to a semiquantitative scale from negative to 3+ (−: 0%; +: 1-33%; ++: 34-66%; +++: 67-100%).

## SUPPLEMENTARY MATERIAL FIGURE


