# Supplementary material for: Fasting cycles potentiate the efficacy of gemcitabine treatment in in vitro and in vivo pancreatic cancer models
Source: Oncotarget. 2015 May 19;6(21):18545–57. doi: 10.18632/oncotarget.4186 (PMC4621909; doi:10.18632/oncotarget.4186)
Supplement: Supplementary file 1 [file oncotarget-06-18545-s001.pdf]

Fasting cycles potentiate the efficacy of gemcitabine treatment in *in vitro* and *in vivo* pancreatic cancer models

Supplementary Material

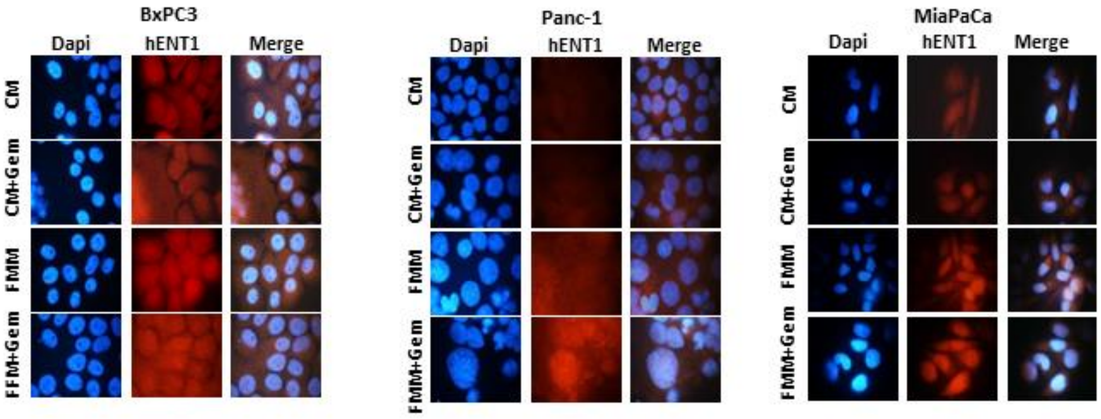

Supplementary Figure 1: hENT1 expression by immunofluorescence in control cells or treated with fasting +/- gemcitabine.
